# The Role of Continuing Professional Training or Development in Maintaining Current Employment: A Systematic Review

**DOI:** 10.3390/healthcare11212900

**Published:** 2023-11-03

**Authors:** Rahman Shiri, Ashraf El-Metwally, Mikael Sallinen, Marjaana Pöyry, Mikko Härmä, Salla Toppinen-Tanner

**Affiliations:** 1 Finnish Institute of Occupational Health, 00032 Helsinki, Finland; mikael.sallinen@ttl.fi (M.S.); marjaana.poyry@ttl.fi (M.P.); mikko.harma@ttl.fi (M.H.); salla.toppinen-tanner@ttl.fi (S.T.-T.); 2 College of Public Health and Health Informatics, King Saud Bin Abdulaziz University for Health Sciences, Riyadh 14611, Saudi Arabia; ashraf.elmetwally@gmail.com; 3 The Health Sciences Unit, Faculty of Social Sciences, Tampere University, 33720 Tampere, Finland

**Keywords:** education, employment, on-the-job training, personnel turnover, return to work, work engagement

## Abstract

The impact of continuing job education and professional development on early exit from the labor market is unclear. This systematic review examined how continuing job education or professional development influences the retention of current employment. We searched the PubMed and Embase databases from their start dates to January 2023. Two reviewers screened the full texts of relevant reports and assessed the methodological quality of the included studies using the adapted Effective Public Health Practice Project quality assessment. We qualitatively synthesized the results of the included studies. We screened 7338 publications and included 27 studies consisting of four cohort and 23 cross-sectional studies in the review. The participants of the selected studies were mostly from the health sector (24 studies). There were 19 studies on staying or leaving a current job, six on employee turnover intention, two on job change, one on return to work, one on early retirement, and one on employment. Continuing employee development or training opportunities were associated with increased intention to stay in a current job, decreased intention to leave a current job, decreased employee turnover intention, job change, or early retirement and with faster return to work. One of the two studies that examined the role of age showed that continuing employee development is a more important factor for retaining current employment among younger than older employees. A few studies found that job satisfaction and commitment fully mediated the relationship between employee development and employee intention to leave current employment. This study suggests that participating in professional training/development is related to a lower risk of leaving current employment.

## 1. Introduction

Employee exit from the labor market is influenced by various factors, such as personal, work, and organizational factors [1]. Age is a key factor that affects the risk of disability retirement, which is higher among older workers [1,2], and the intention to quit the current job, which is lower among older workers [3]. Education level also plays a role, as workers with lower education are more likely to leave the labor force due to disability, unemployment, or early retirement [1,4], while workers with higher education are more likely to change their current job [3,5]. Work-related factors, such as workload, working conditions, work–life balance, and burnout, affect employees’ intention to leave their job [3,6,7]. Psychological and organizational factors, especially low job control, are associated with disability retirement [8]. On the other hand, interventions such as adjusted job demands, social support at work, coaching, and job training can reduce the rate of premature exit from the labor market in workers with a chronic disease [1].

To keep and enhance their professional competence (knowledge and skills), workers need to engage in continuing professional development. This also helps them advance their careers, practice safely, provide better services to clients, and maintain consumer trust [9,10,11]. Continuing professional development is more common among health care workers [9], while its benefits for other occupations are less explored. Health care workers participate in continuing professional education and training to develop their careers, stay updated, and improve the quality of patient care [7,12].

Continuing professional development covers various short courses, conferences, workshops, seminars, and other short training programs. It can have different impacts on health professionals, such as increasing clinical knowledge; fostering networking and collaboration; changing attitudes; enhancing skills, competence, and performance; and influencing clinical practice [9,13]. By taking part in continuing education and training at work, workers can improve and refresh their skills and learn new ones [14]. On-the-job vocational training improved the mental health, sense of coherence, psychological stress, dysfunctional attitudes, and smoking rate among health care workers [15]. Employees who receive continuing job education or training report higher job satisfaction [16,17,18,19].

However, the effect of professional development and job education or training on staying or leaving the current employment is unclear. The purpose of this systematic review was to investigate how professional development and job education or training are related to maintaining or exiting the current employment. We also examined whether the relationship varies between younger and older workers.

## 2. Methods

### 2.1. Search Strategy

We followed the PRISMA guidelines [20] to design the review protocol. We searched PubMed and Embase from their start dates to 2 January 2023 using a combination of MeSH terms (PubMed), Emtree terms (Embase), and text words (Table 1). We also performed an extra search in Google Scholar. We did not apply any filters on the participants’ age or sex or on the publications’ language. We manually checked the references of the relevant articles on this topic to find more reports that might be useful.

### 2.2. Inclusion and Exclusion Criteria

Using PubMed, Embase, and Google Scholar, the first author searched for reports related to the topic and selected the ones that seemed relevant for further evaluation. Then, two reviewers (R.S. and A.L.-M.) screened the abstracts and full texts of the selected reports independently. The inclusion criteria were studies that investigated the effects of education, training, or job development on work retention or exit from paid employment using randomized or non-randomized controlled trials, cross-sectional, case control, and cohort designs. The exclusion criteria were studies that focused on vocational rehabilitation, employment services, and educational services for job seekers or people with a disability, as these interventions aimed at changing jobs rather than enhancing skills for the current job. Moreover, studies that used an organization as a unit of analysis and reported an employee turnover rate at the organizational level were excluded. Additionally, qualitative studies were not included in the review. The reviewers discussed any disagreements and reached a consensus.

### 2.3. Quality Assessment

The quality of the studies included in this review was evaluated by two independent reviewers (R.S. and A.L.-M.) using an adapted version of the Effective Public Health Practice Project quality assessment tool [21]. This tool assessed five types of bias: selection bias, performance bias, detection bias, attrition bias, and confounding (see Supplementary Table S1). The reviewers discussed and resolved any disagreements about the quality ratings.

### 2.4. Data Synthesis

We extracted the following characteristics from the studies that met the inclusion criteria for the review: study design, publication year, country of origin, study population description, sample age and sex distribution, sample size, professional education or training type, work participation or exit from paid employment, summary results, and confounding factors adjustment. We performed a qualitative synthesis of the results of the included studies because of the heterogeneity in professional training and outcome.

## 3. Results

**Figure 1:**
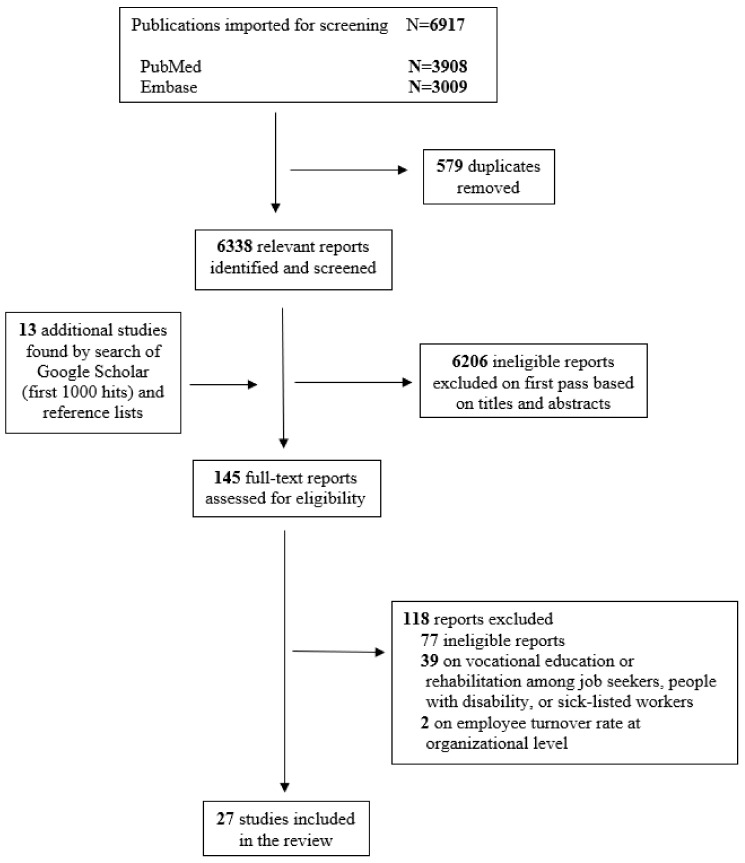
PRISMA flow diagram of the studies selection.

A total of 3908 publications were retrieved from PubMed, and 3009 were retrieved from Embase (Figure 1). The first reviewer removed 579 duplicates and screened 6338 titles and abstracts from PubMed, Embase, and the first 1000 hits from Google Scholar (total: 7338). Google Scholar only allows screening the first 1000 results. Then, two reviewers assessed 145 abstracts or full-text articles for relevance. Out of those, 77 reports were excluded for not meeting the eligibility criteria, and 39 reports on vocational re-education or rehabilitation among job seekers or people with a disability and two studies on employee turnover rate were omitted from the review because they only reported the outcome at the organizational level and not at the individual level. Finally, the review included 27 studies consisting of four cohort studies and 23 cross-sectional studies. The participants of the selected studies were diverse but mostly from the health sector. Out of the 27 studies, 22 involved health care workers as the target population, while 2 focused on faculty members of health or medical sciences. The remaining three studies included people with a chronic disease and bank staff as participants.

The studies were published in different time periods. Eight studies were published between 2001 and 2010, eight were published between 2011 and 2015, and 11 were published between 2016 and 2022. The studies were conducted in various countries. Australia [16,22,23] and China [5,24,25] had three studies each. New Zealand [6,26], the United Kingdom [7,12], and the United States [27,28] had two studies each. Canada [29], Denmark [30], Ethiopia [31], Finland [32], Ghana [33], Japan [34], Italy [35], Pakistan [36], South Korea [37], Sweden [38], Taiwan [39], and the Netherlands [1] had one study each. One study recruited participants from Singapore and the USA [40], one study recruited participants from eight European countries (Belgium, Finland, France, Germany, Italy, Poland, Slovakia, and the Netherlands) [41], and another study was conducted in seven sub-Saharan African countries (Ethiopia, Kenya, Nigeria, Rwanda, Tanzania, Uganda, and Zambia) [42]. The number of participants in the included studies varied from 81 to 88,948.

The effects of professional development or training on job retention or turnover were assessed in three studies [27,28,30] using administrative data and 24 studies using self-reported data (Table A1 and Table S2). The risk of selection bias was low in six studies, moderate in 11 studies, and high in 10 studies (Supplementary Table S2). Eighteen studies adjusted for some or all confounding factors. Attrition bias was low in all studies except two.

### 3.1. Job Retention

Five studies investigated the relationship between professional development or training and staying at the current job or intending to do so. An eight-year cohort study [28] reported that junior faculty members who participated in a development program were 11% more likely to remain at the same job than non-participants (67% vs. 56%, *p* = 0.04). Additionally, cross-sectional studies showed that professional development opportunities were linked to a higher intention to stay at the current job [16,22,29,42]. Professional development or a training opportunity was the main motivator for staying at a current job, and 80% of laboratory professionals from seven sub-Saharan African countries rated it as the most important or a very important factor for job retention [42]. Younger employees valued continuing professional development more than older employees for staying at the current job [22].

### 3.2. Leaving a Job

Four studies examined the association between professional development or training and leaving a job or the workforce, and 10 studies examined the intention to leave a job (Table A1). A large cohort study [27] found that women who attended 4-day early- and mid-career faculty professional development programs were less likely to leave their job than women who did not attend the programs. The programs had a positive effect on women’s job retention, as those who participated more than once were less likely to quit than those who only joined once. A similar finding was reported in a cohort study [12] in which employees who left their first job within six months cited a lack of study days (40%) and other courses (43%) as important factors in their decision. A cross-sectional study found that patients with rheumatoid arthritis who received additional job training after their diagnosis were less likely to leave the workforce than those who did not (adjusted OR 0.5, 95% CI 0.4–0.8) [1]. Dissatisfaction with development opportunities was also a major reason for nurses to leave their job in 51.4% of those who had left their institution [41].

Furthermore, a lack of professional development opportunities [31,32,33] and low perceived investment in employee development [39,40] increased the intention to leave a job. Some of the factors that influenced this intention were a lack of access to professional development [23,38], a lack of study opportunities [23], and a lack of access to courses other than study days [12] and/or study days [12]. A lack of professional opportunities ranked second after a low salary as a reason for leaving nursing care, and this was consistent between nurses aged < 45 years and those aged ≥ 45 years [38]. However, a cross-sectional study showed that a lack of career advancement and mandatory continuing professional development did not affect the intention to leave the dental nursing profession [7]. Another study also found no direct or indirect association between professional development and intention to leave an organization or profession through burnout and work engagement [6].

The relationship between the perceived investment in employee development and employee intention to a leave job was mediated by different factors in two studies [39,40]. Job satisfaction and affective commitment fully explained the relationship between perceived investment in employee development and the intention to leave a job for nurses [40]. For health care professionals in underserviced areas with a government subsidy program, the relationship between perceived investment in employee development and the intention to leave a job was fully explained by employee professional and organizational commitment, while for those without a government subsidy program, there were both direct and indirect effects of perceived investment in employee development on the intention to a leave job [39].

### 3.3. Turnover Intention

As shown in Table A1, six studies examined the relationship between turnover intention and professional development. Employees who had domestic training or overseas study outside of work had a turnover intention of 46%, while those who did not have any domestic training or overseas study had a turnover intention of 68% [25]. Several factors related to professional development, such as limited opportunities [24,35], inadequate continuing education [5], dissatisfaction with professional development [37], and low perceived investment in employee development [36] were associated with increased turnover intention. The effect of professional development opportunities on turnover intention differed by gender and profession [5,24]. A higher intention to leave the job was linked to inadequate professional development opportunities for men but not for women [24]. Similarly, doctors who had enough opportunities for continuing professional education had a lower intention to leave, but this was not the case for nurses [5].

Continuing professional education did not affect turnover intention for rural healthcare workers [5]. Moreover, the effect of satisfaction with professional development on turnover intention varied according to length of employment for nurses [37]. Satisfaction with professional development reduced turnover intention for nurses who had been employed for 13 to 18 months but not for those who had been employed for less than 12 months [37]. Training to improve skills or competences reduced turnover intention for nurses who had been employed for less than 6 months, while opportunities for professional development reduced turnover intention for nurses who had been employed for 7 to 24 months [35]. One study investigated the mechanisms underlying the link between perceived investment in employee development and turnover intention [36]. It found that job satisfaction and affective commitment fully mediated this link [36].

### 3.4. Return to Work, Job Change, Early Retirement, and Employment

Five studies were reviewed on different aspects of career transitions among workers (Table A1). A Danish study examined the effect of wage-subsidized job training on the duration of return to work and subsequent employment among sick-listed workers. The study found that the intervention shortened the time to return to non-subsidized work by three weeks but did not affect the stability of the subsequent employment [30]. Another study surveyed psychiatrists who moved or did not move to another area; it reported that professional support and development was a key factor in their decision to move to another area for 44% and 47% of them, respectively [26]. A third study investigated the motives for changing a job in the past five years among laboratory professionals from seven sub-Saharan African countries and revealed that the main reasons were lack of professional development or training (27.8%), lack of benefits (23.5%), relocation (22.6%), and poor working conditions (13.0%) [42]. A fourth study that analyzed the rate of early retirement among employees with different levels of domestic off-the-job training and/or overseas study showed that it was lower for those with some training or study (44%) than for those with none (63%) [25]. Lastly, the only study that investigated the relationship between professional development and employment status reported that participants who underwent training to enhance their professional skills had a higher probability of being employed than unemployed [34].

## 4. Discussion

The main finding of this systematic review is that there is a positive relationship between professional development or training and work participation. Employees who engage in skill development or training are more likely to stay in their current job than those who do not. However, the quality of the evidence is low, as 85% of the studies used a cross-sectional design, and more than a quarter of the studies did not adjust for confounding factors.

A review of the literature revealed that older nurses (over 45 years) have less access to continuing professional learning and development [43]. Only two cross-sectional studies examined the effect of age on the link between professional skill development and work participation [22,38]. One study found that continuing professional development was more important for retaining younger health professionals (mean age: 35.6 years) than older health professionals (mean age: 40.2 years) working with people with a disability in rural areas [22]. For younger health professionals, professional support, continuing professional development, and a high autonomy of practice were the main factors for staying in their current job, while for older health professionals, travel arrangements and a high autonomy of practice were the main factors. The other study reported that a lack of opportunities for professional development was a reason for leaving nursing care among both nurses younger than 45 years and those aged 45 years or older [38]. However, nurses younger than 45 years were more likely to leave nursing care due to the nursing workload and a low salary than older nurses aged ≥ 45 years [38]. Additionally, a qualitative study of 84 nurses over 50 years [44] that was not included in this review suggested that part-time work, flexible working hours, and continuing professional development could increase work participation. The relationship between continuing professional education and work retention among older workers has not been well studied. Future research using quasi-experimental and prospective cohort designs could help to determine if ongoing job training lowers the risk of leaving the workforce among ageing workers.

**Figure 2:**
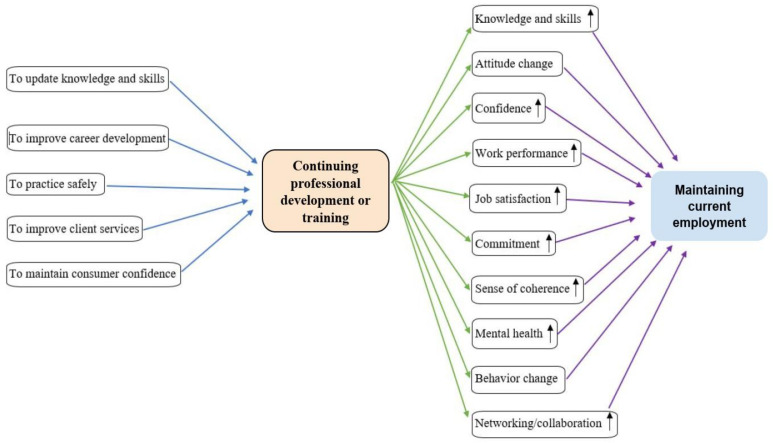
A conceptual diagram showing the potential mediators of the association between continuing professional development or training and maintaining current employment.

There is limited evidence on the mechanisms that explain how continuing professional education/training influences staying in a current job. Professional training is linked to higher organizational commitment [45]. Job satisfaction and affective commitment [40] or professional and organizational commitment [39] fully mediate the relationship between perceived investment in employee development and intention to quit a current job. Moreover, the association between perceived investment in employee development and employee turnover intention was fully mediated by job satisfaction and affective commitment [36]. However, one study did not find any indirect relationship between professional development and the intention to leave a profession through reducing burnout or increasing work engagement [6]. In addition to commitment and job satisfaction, continuing professional education enhances employees’ knowledge, skills, confidence, sense of coherence, work performance, and mental health, and it leads to changes in attitude, behavior, and practice [9,13]. These positive outcomes of continuing professional education can increase employee retention (Figure 2). Continuing professional education is related to higher career development and job satisfaction among workers [16,17,18,19,42], and to less work–family conflict, family complaints, and guilt regarding family [25]. Job satisfaction promotes employee retention [3,16], and job dissatisfaction is a common reason for quitting a job [23] or planning to retire early [46]. Mid-career physicians (41–60 years old) reported a lack of professional satisfaction as a more important factor influencing their retirement intention than physicians older than 60 years [46]. Previous studies have shown mixed results on the relationship between continuing professional training and work engagement. One study reported that workers who received professional training were more engaged in their work than workers without training [25], while another study found no link between continuing professional development and work engagement [6].

This review indicates that participating in professional training is linked to a lower risk of leaving current employment. However, continuing professional development opportunities are scarce, especially in remote and rural areas [5,7,26]. A major obstacle to accessing continuing professional development is a lack of financial support [14,47], and some workers have to pay for their own professional training [7]. Moreover, organizations reduce their budgets for professional learning and development during economic crises [48]; for example, the satisfaction rate with access to continuing professional development among rural Australian health care workers declined from 70% in 2005 to 35% in 2008 [49].

The current review has some limitations. Out of the 27 studies that explored this topic, most of them had a cross-sectional design (23 studies) and a small sample size. Only four studies had a cohort design, and a third of the studies did not adjust the observed associations for any confounding factor. In addition, few studies investigated the mechanisms and factors that influenced the effect of professional development/training on staying in a current job or in the labor market.

Future research should adopt more rigorous methods, such as quasi-experimental and longitudinal designs, to evaluate the impact of different types of training on the likelihood of exiting the labor force, especially among older workers. Future research should also identify which aspects of professional development and training are more effective or relevant for job retention than others (e.g., pedagogical aspects and personalization). Furthermore, in future research, it would be useful to differentiate between employees who leave their current job for a better one (qualitative employability) and those who leave for any other reason (quantitative employability) [50].

## 5. Conclusions

This review indicates that engaging in continuing professional training or development may help workers to retain their current employment. However, more high-quality studies, especially among older workers, are needed to examine the role of continuing job training in preventing labor-force exit.

## Figures and Tables

**Table 1:** PubMed and Embase searches conducted on 2 January 2023.

Search	Query	No. of Items Found
PubMed		
#1	professional training[tiab]OR retraining[tiab] OR professional learning[tiab] OR relearning[tiab] OR reeducation[tiab] OR re-education[tiab] OR “education, professional, retraining”[Mesh] OR “vocational education”[Mesh] OR professional education[tiab] OR professional development[tiab] OR “education, continuing”[Mesh] OR continuing education[tiab] OR “interprofessional education”[Mesh] OR “inservice training”[Mesh] OR “staff development”[Mesh] OR job development[tiab] OR employee development[tiab] OR employees’ development[tiab] OR workplace learning[tiab] OR workplace training[tiab]	119,953
#2	work engagement[Mesh] OR “work engagement”[tiab] OR “employee participation”[tiab] OR “work participation”[tiab] OR “career participation”[tiab] OR “labor participation”[tiab] OR “labour participation”[tiab] OR “labor market participation”[tiab] OR “labour market participation”[tiab] OR employment[Mesh] OR unemployment[Mesh] OR return to work[Mesh] OR “return to work”[tiab] OR disability pension[tiab] OR disability retirement[tiab] OR retirement[Mesh] OR pensions[Mesh] OR early retirement[tiab] OR retired early[tiab] OR workforce recruitment[tiab] OR “workplace engagement”[tiab] OR workability[tiab] OR work ability[tiab] OR labor market exit[tiab] OR labour market exit[tiab] OR exit from employment[tiab] OR “personnel turnover”[Mesh]	127,609
#3	#1 AND #2	5162
#4	#3 Filters: Biography, case reports, comment, guideline, lecture, legal case, legislation, letter, editorial, news, newspaper article, portrait, published erratum, retracted publication, review, books and documents, case reports, dictionary, duplicate publication	665
#5	#3 NOT #4	4497
Final	#5 Filters: Humans	3908
Embase		
#1	‘interprofessional education’/exp OR ‘retraining’/exp OR ‘training’/mj OR ‘learning’/mj OR ‘skill retention’/exp OR ‘professional training’ OR ‘professional learning’ OR ‘relearning’ OR ‘reeducation’/exp OR ‘reeducation’ OR ‘re-education’ OR ‘vocational education’/exp OR ‘mentoring’/exp OR ‘lifelong learning’/exp OR ‘interdisciplinary education’/exp OR ‘in service training’/exp OR ‘continuing education’/exp OR ‘continuing education’ OR ‘adult education’/exp OR ‘refresher course’/exp OR ‘professional development’/exp OR ‘staff development’ OR ‘job development’ OR ‘employee development’ OR ‘employees development’ OR ‘workplace learning’ OR ‘workplace training’	228,705
#2	‘work engagement’/exp OR ‘work engagement’ OR ‘employee participation’ OR ‘work participation’ OR ‘career participation’ OR ‘labor participation’ OR ‘labour participation’ OR ‘labor market participation’ OR ‘labour market participation’ OR ‘employment’/exp OR ‘employment’ OR ‘unemployment’/exp OR ‘unemployment insurance’/exp OR ‘unemployment’ OR ‘return to work’/exp OR ‘return to work’ OR ‘disability pension’/exp OR ‘disability pension’ OR ‘disability retirement’ OR ‘retirement’/exp OR ‘early retirement’ OR ‘retired early’ OR ‘workforce recruitment’ OR ‘workplace engagement’ OR ‘workability’ OR ‘work ability’ OR ‘labor market exit’ OR ‘labour market exit’ OR ‘exit from employment’ OR ‘turnover rate’/exp OR ‘turnover rate’	251,313
#3	#1 AND #2	4289
#4	#3 AND (‘editorial’/it OR ‘letter’/it OR ‘note’/it OR ‘review’/it)	638
#4	#3 NOT #4	3651
Final	#5 AND ‘human’/de	3009

## Supplementary Materials

The following supporting information can be downloaded at: https://www.mdpi.com/article/10.3390/healthcare11212900/s1, Table S1: Adapted Effective Public Health Practice Project quality assessment; Table S2: Risk of bias assessment.

## Appendix A

**Table A1:** Characteristics of studies included in the review, which are presented according to the design of the studies in order of their publication year.

Study (Authors and Year of Publication)	Country	Population	Sex Distribution	Mean Age (or Age Range) at Baseline	Number of Participants (Included in the Analysis)	Professional Training	Outcome	Results	Adjustment for Confounders
Cohort studies									
Holm et al., 2017 [30]	Denmark	Workers in non-subsidized employment on a sick leave spell longer than four weeks; follow-up from 2008 to 2011	55.6% of those who did not receive education or training and 53.3% of those who received training were women.	Mean age of 38.2 ± 10.3 for participants who did not receive education or training and 37.8 ± 10.3 for those who received training.	88,948	Non-formal education (e.g., shorter courses), wage-subsidized internships, and wage-subsidized job training	The duration of returning to non-subsidized employment and the duration of the subsequent employment	Job training had the largest effect on employment. It shortened the duration until returning to non-subsidized employment by three weeks but had no effect on duration of the subsequent employment spell. Non-formal education and subsidized internships had no beneficial effects on employment.	Age, sex, education, number of children, migration background, employment sector, medical condition, unemployment rate, and commuting area
Chang et al., 2016 [27]	USA	Female faculty members from the Association of American Medical Colleges; follow-up from 1988 to 2009	Women (two control groups: women and men)	Mean age of 41.1 ± 5.2 years for women who attended the program and 43.2 ± 6.7 for women who did not	3268 women attended the program and 17,834 women and 40,319 men who did not attend the program.	National career development program based on register data and a 4-day early- and mid-career faculty professional development program to provide academic career skills for early- and mid-career faculty	Leaving job	Women who attended the program less frequently left academic medicine than women and men who did not attend the programs. Women who participated in more than one program less often left academic medicine than women who participated in one program. When comparing women who attended the program with women who did not attend the programs, the hazard ratio was 0.85 (95% CI 0.74–0.98) for assistant professors, 0.76 (95% CI 0.64–0.93) for associate professors, and 0.68 (95% CI 0.50–0.92) for full professors for leaving their job.	Age, tenure track status, degree type, and department type
Ries et al., 2012 [28]	USA	Assistant professors in health sciences	52.2% of program participants and 51.5% of matched non-participants were women.	Not reported	113 program participants and 202 matched non-participants of the program.	A 7-month development program for junior faculty. It included professional development workshops, career planning, individualized academic performance counseling, mentoring with a senior faculty member, and network building with other faculty.	Staying at current job	67% of program participants and 56% of matched non-participants stayed in their job at the end of the eight-year probationary period (*p* = 0.04).	Matched for gender, academic series (research vs. clinical), initial academic rank (academic experience), hire date, and department. Adjusted for gender and ethnicity.
Robinson and Tingle 2003 [12]	UK	Mental health nurses qualifying from diploma course	70% were women	60% were aged < 30 years, and 40% were aged ≥ 30 years.	444	Continuing professional development consisted of continuing education (degrees, other post-registration courses, and study days and workshops).	Leaving job	Of those (n = 30) who left their first job within 6 months after qualifying, 40% regarded lack of opportunities to go on study days as an important reason for leaving, and 43% regarded lack of opportunities to go on courses other than study days as an important reason for leaving. Intention to leave first job: dissatisfaction with continuing education opportunities was listed as reason for considering leaving a job. 8% considered lack of opportunities to start courses other than study days (third most common reason of dissatisfaction), and 6% considered lack of opportunities to attend study days (eighth most common cause of dissatisfaction).	Unadjusted
Cross-sectional studies									
Wang et al., 2022 [25]	China	Physicians from seven provinces	43.4% were women	29% were aged ≤ 34 years, 34.7% were aged 35–44 years, and 36.3% were aged ≥ 45 years.	3182	Number of domestic off-the-job trainings of more than 3 months in the last 5 years. The responses were dichotomized (yes vs. no);overseas study (visiting scholarships).	Turnover intention and early retirement	Turnover intention was 45.6% in those with training and 68.4% in those with no training. Turnover intention: odds ratio 0.87 (95% CI 0.69–1.10) for domestic training only, 0.90 (95% CI 0.65–1.25) for overseas study only, and 0.54 (95% CI 0.38–0.77) for both domestic and overseas training compared with no domestic and overseas training.Early retirement was 43.7% in those with training and 62.9% in those with no training. Early retirement: odds ratio 0.87 (95% CI 0.70–1.08) for domestic training only, 0.67 (95% CI 0.49–0.91) for overseas study only, and 0.63 (95% CI 0.45–0.89) for both domestic and overseas training.	Sex, age, marital status, education level, economic status, hospital level, hospital type, academic status, physician specialty, ratio of physicians to beds, work pressure, pay justice, task justice, patient trust, unreasonable request by patients, and family support
Miura et al., 2021 [34]	Japan	Members of three alumni associations of a dental hygiene training school	Women only	20–59 years, mean age 39.1 ± 8.9 years	537	Participation in professional skill development training programs in the past year (yes vs. no)	Employed vs. unemployed as a dental hygienist	Participation rate in professional skill development training was 54.9% among 366 employed participants and 12.9% among 171 unemployed participants. Adjusted OR: 6.50 (3.82–11.07);	Age, marital status, having children, priority assigned to wages, and priority assigned to working hours
Gan et al., 2020 [24]	China	General practitioners	63.8% were women	Mean age 37.4 ± 7.9 years	3236	Professional development opportunities: (1) few, (2) general, and (3) more	Turnover intention. A 6-item turnover intention scale (score 0 to 24) was used.	7.3% reported professional development opportunities. Compared with general practitioners with few professional development opportunities, turnover intention was lower in those with general (estimate: −0.43, *p* = 0.003) or more (estimate: −0.68, *p* = 0.015) opportunities.In a gender-specific analysis, limited professional development opportunities were associated with a higher level of turnover intention among men but not among women. The estimates for men were −0.70 (*p* = 0.005) for general opportunities and −1.33 (*p* = 0.004) for more opportunities.	Age, sex, marital status, education, income, professional title, type of contract, region, overtime work, work hours per week, professional identify, work stress, management responsibility, night work, job satisfaction, and work tenure in general practice
Liu and Mao 2020 [5]	China	Rural healthcare workers (doctors and nurses)	69.1% were women	36.6% were younger than 30 years, 35.6% were aged 30–39 years, and 27.8% were aged 40 years or older.	4118 (2490 doctors and 1628 nurses)	Continuing medical education (educational and training activities to maintain or improve employee knowledge, skills, and performance). Opportunities, participation, and expectation of continuing medical education were studied.	Turnover intention	8.8% reported sufficient opportunities for continuing medical education. Participation and expectation of continuing medical education were not associated with turnover intention in both doctors and nurses. Sufficient opportunities for continuing medical education were associated with a higher prevalence of lack of employee turnover intention (odds ratio 1.70, 95% CI 1.26–2.28). The association was found among doctors (OR 2.18, 95% CI 1.42–3.34) but not among nurses (OR 1.22, 85% CI 0.79–1.87).	Age, sex, marital status, level of education, technical job title, income, and type of rural healthcare organization
Du et al., 2019 [39]	Taiwan	Health care professionals working in underserviced areas	76.5% were women	Most of the participants were aged 30–50 years.	616	Perceived investment in employee development (perception of organization’s commitment to maintain and improve employee skills and competencies). A 9-item questionnaire was used to assess skill training, career counseling, and organizational support.	Intention to leave job	Perceived investment in employee development was associated with lower intention to leave job. Employee professional and organizational commitment fully mediated the relationship between perceived investment in employee development and intention to leave job in employees with government subsidy, but there were both direct and indirect effects in those without government subsidy program	Government subsidy and commitment
Kols et al., 2018 [31]	Ethiopia	Anesthetists working full-time in public-sector hospitals	25.1% were women	61.2% were aged ≤ 30 years and 38.8% were aged > 30 years.	251	Limited opportunities for professional development consisted of (1) poor access to higher education, (2) limited opportunities for promotion, and (3) limited opportunities for in-service training.	Intention to leave job in the next year	Limited professional development opportunities were associated with intention to leave job. Adjusted OR: 1.91 (95% CI 1.26–2.90) for yes/no; limited professional development opportunities.	Age, living conditions, work burden, conditions at workplace, and type of hospital (district, regional, or referral) Sex, marital status, education, years of public health service, relationship with supervisor and co-workers, compulsory service obligation, and basic salary did not remain significant in a stepwise regression analysis
Moloney et al., 2018 [6]	New Zealand	A sample of registered nurses	93.9% were women	18–75, mean age of 48.8 ± 11.5 years	2876	Professional development was measured by two items: (1) I am able to take time off for training, and (2) I am able to keep up with developments to do my job.	Intention to leave the organization and intention to leave the profession. Each outcome was measured with three items.	Professional development was neither directly nor indirectly associated with intention to leave organization or profession through reducing burnout and increasing work engagement.	Workload, emotional demands, work–life interference, supervisor support, colleague support, organizational support, autonomy, and self-efficacy
Nowrouzi et al., 2016 [29]	Canada	Registered nurses	94.8% were women	Mean age of 48.0 ± 10.5 years	459	The importance of staff development in the organization (opportunity for job advancement in the workplace; yes vs. no).	Intention to remain in current job for the next five years	Adjusted odds ratio: 3.04 (95% CI 1.13–8.13)	Sex, age, marital status, educational level, employment status, living cost, workload allocation, years of nursing experience, number of hours worked per week, number of overtime hours worked per week, and northeastern Ontario lifestyle
Yu and Kang 2016 [37]	South Korea	Nurses in their first 18 months of employment	Not reported	20–33 years; mean age of 23.4 ± 2.4 years; 86.3% aged 25 years or younger	443	Satisfaction with professional development (score 1 to 4; higher scores indicated higher satisfaction)	Turnover intention. It was measured using a 4-item scale and was dichotomized as high vs. low.	In the total sample, there was an inverse correlation between satisfaction with professional development and turnover intention (correlation: −0.198, *p* < 0.001). Among nurses with 0 to 6 months of employment, satisfaction with professional development did not differ between nurses with low and high turnover intention (mean: 2.82 vs. 2.81, *p* = 0.83) in an unadjusted analysis. However, after adjustment for other factors, the adjusted regression coefficient was positive (β = 0.47, *p* = 0.002), showing a positive association between satisfaction with professional development and turnover intention. Among nurses with 7 to 12 months of employment, satisfaction with professional development did not differ between nurses with low and high turnover intention. Among nurses with 13 to 18 months of employment, satisfaction with professional development was lower in nurses with high turnover intention compared with nurses with low turnover intention (unadjusted mean: 2.42 vs. 2.83, *p* < 0.001; adjusted regression coefficient: −0.48, *p* = 0.001).	Stepwise regression was used, and the following characteristics were considered: age, marital status, education, work type, work schedule, desired hospital, orientation period, becoming part of a team, and practice support.
Agyapong et al., 2015 [33]	Ghana	Community mental health workers	Not reported	Not reported	164	Lack of opportunities for professional development	Intention to leave job	37.2% of participants reported intention to leave the profession for reasons other than stigma and risk. Lack of opportunities for professional development was one of several listed reasons.	Unadjusted
Gallego et al., 2015 [22]	Australia	Health professionals working with people with a disability in rural areas	93.9% were women	Mean age of 36.0 ± 11.7 years	165	Opportunity for continuing professional development: minimal, adequate, ideal	Staying at current job	Participants with adequate or ideal opportunity for continuing professional development were more likely to stay in their current job than those with minimal opportunity. Adequate access to professional development was the third contributor to maintaining their current job after high autonomy of practice and travel arrangements. Professional support, continuing professional development, and a high autonomy of practice were the most important contributors to maintaining a current job in younger participants (mean age: 35.6 years) with a lower household income and not having children, while travel arrangements and a high autonomy of practice were the most important contributors to maintaining a current job in older participants (mean age: 40.2 years) with a high household income and having children.	Age, household income, and having dependent children
Tomietto et al., 2015 [35]	Italy	Nurses in their first 24 months of employment	79.5% were women	Mean age of 31.0 ± 8.2 years	156	Competence acquisition (training to improve skills or competences) and future prospects (opportunities for professional development)	Turnover intention It was measured using a 4-item scale.	Competence acquisition was inversely associated with turnover intention among nurses in their first 0–6 months of employment (β = −0.42, *p* < 0.01) but not among nurses in their first 7–12 or 13–24 months of employment.Opportunities for professional development were inversely associated with turnover intention among nurses in their first 7–12 (β = −0.39, *p* = 0.01) or 13–24 (β = −0.30, *p* = 0.05) months of employment but not among nurses in their first 0–6 months of employment.	Co-worker support, formal understanding, informal understanding, competence acquisition (for professional development), and professional development (for competence acquisition)
Chenoweth et al., 2014 [16]	Australia	Registered and enrolled nurses caring for older persons or persons with dementia	76% were women	20% were aged < 35 years, 58% were aged 30–50 years, and 22% were aged > 50 years.	3983	Training and study opportunities	Intention and motivation to remain in the current job	57% of participants strongly or somewhat agreed that training, career, and study opportunities influence the retention of nurses. The coefficient of nurse retention was 0.66 for training and study opportunities.	Unadjusted
Marinucci et al., 2013 [42]	Ethiopia, Kenya, Nigeria, Rwanda, Tanzania, Uganda, and Zambia	Medical laboratory professionals	40.2% were women	20–64 years; mean age of 34 years. 8% were younger than 25 years, and 8.5% were aged 50 years or older.	224	Opportunity for professional development or training	Changing job in the past five years and staying at current job	57% of laboratory professionals changed their jobs at least once in the past five years. Lack of professional development or training was the leading motive for changing a job (27.8%), followed by a lack of benefits (23.5%), relocation (22.6%), and poor working conditions (13.0%). Opportunity for professional development or training was the leading incentive to maintain the current job. 80% of participants rated opportunity for professional development or training as the most important or very important contributor to job retention.	Unadjusted
Turner et al., 2012 [7]	UK	Dental nurses	All were women	19–65 years; mean age of 38.2 ± 10.7 years	267	Mandatory continuing professional development	Intention to leave dental nursing profession	Mandatory continuing professional development was associated with an intention to leave the dental nursing profession in a univariable analysis. However, the association disappeared after adjustment for covariates. Lack of opportunities to progress in the job was statistically significantly associated with an intention to leave the dental nursing profession.	Age, physical working conditions, remuneration, hours of work, freedom to choose work method, recognition for good work, amount of variety in job, lack of opportunities to progress, support for keeping up to date, support for training in work area, support for developing expertise, general principle of registration, problem in funding continuing professional development, employer’s unfavorable view of continuing professional development, and level of registration fee
Malik et al., 2011 [36]	Pakistan	Tellers of private sector banks	4% were women	23% were aged younger than 35 years, 58% were aged 35–45 years, and 19% were aged older than 45 years.	177	Perceived investment in employee development. It was measured using a 9-item scale.	Turnover intention	Perceived investment in employee development was associated with a lower turnover intention (correlation −0.43, *p* < 0.01). The association between perceived investment in employee development and turnover intention was fully mediated by affective commitment and job satisfaction.	Affective commitment and job satisfaction
Estryn-Behar et al., 2010 [41]	Belgium, Finland, France, Germany, Italy, Poland, Slovakia, and the Netherlands	Nurses working in hospitals, nursing homes or home care institutions who left their institutions	90.4% were women	Mean age of 38.95 ± 11.7 years	941	Dissatisfaction with development opportunities	Leaving the institution	Dissatisfaction with development opportunities was considered as a contributor to a large extent to the decision to leave the institution in 51.4% of nurses who had left their institution. It was the 6th most common reason for leaving the institution after (1) time pressure and quality of care, (2) dissatisfaction with use of one’s competence and a lack of autonomy, (3) dissatisfaction with pay, (4) relationship problems, and (5) insufficient staff numbers.	Unadjusted
Flinkman et al., 2008 [32]	Finland	Registered nurses	93.2% were women	24–29 years; mean age of 26.8 ± 1.45 years	147	Opportunities for professional development as measured using four items	Intention to leave the profession	Poor opportunities for professional development were associated with an intention to leave the profession.	Unadjusted
Garrett et al., 2008 [23]	Australia	Pharmacy staff (pharmacists, pharmacy graduates, pharmacytechnicians, pharmacy assistants, and clerical staff)	Not reported	38% were aged 25–34 years, 19% were aged 35–44 years, 24% were aged 45–54 years, and 16% were aged ≥ 55 years.	81	Professional development	Intention to leave job within two years	18 participants intended to leave the health service profession within the next two years. A lack of access to professional development was listed as one of the reasons for leaving employment. Nine participants reported that their job expectations had not been met. Six of them (67%) intended to leave their job within the next two years. A lack of access to professional development and a lack of study opportunities were listed as reasons for leaving the job within the next two years	Unadjusted
Fochsen et al., 2005 [38]	Sweden	Registered or assistant nurses who left nursing care	84% were women	Mean age of 41.6 years; 66% were aged < 45 years, and 34% were aged ≥ 45.	158	Inadequate opportunities for professional development	Intention to leave nursing care	A lack of professional opportunities was listed as the second most important reason for leaving nursing care after an unsatisfactory salary. There was no difference between nurses aged < 45 years and those aged ≥ 45.	The association did not differ between men and women, younger and older nurses, or assistant and registered nurses.
Lau et al., 2004 [26]	New Zealand	Actively practicing vocationally registered psychiatrists	35.8% were women	19.7% were aged ≤ 40 years, 69.2% aged were 41–60 years, and 5.1% were aged > 60 years.	157	Availability of professional support and development	Intention to change workplace	71% of psychiatrists listed professional support and development as an important or very important factor in choosing their practice area, and 47% listed it as an important or very important factor that might make psychiatrists move to another area. Of psychiatrists who previously moved (n = 36), 44% listed professional support and development as an important or very important factor in making the decision to move to another area. Metropolitan psychiatrists rated professional development as a slightly less importantreason for leaving than non- metropolitan (43% vs. 62%).	Unadjusted
Lee and Bruvold 2003 [40]	Singapore and the USA	Nurses	100% of participants from Singapore, and 99% of those from the USA were women	Mean age of 41.6 years for sample from the USA and 29.8 years for sample from Singapore. 72.7% of participants from Singapore and 5% of those from the USA were aged 30 years or younger, and 27.3% of participants from Singapore and 95% of those from the USA were older than 30 years.	405 (230 from the USA and 175 from Singapore)	Perceived investment in employee development. It was assessed using a 9-item scale.	Intention to leave job as measured using three items	Perceived investment in employee development was inversely correlated with an intention to leave a job in both studies. There was no direct association between perceived investment in employee development and an intention to leave a job. The association was fully mediated by job satisfaction and affective commitment.	Job satisfaction, continuance commitment, and affective commitment
Chorus et al., 2001 [1]	The Netherlands	People with rheumatoid arthritis	70.1% of patients were withdrawn from the labor force, and 56.1% of those in paid employment were women.	20–59	720	Additional job training after diagnosis of rheumatoid arthritis	Withdrawal from the labor force	Patients who completed additional job training withdrew from the labor force less often than those who did not. Adjusted odds ratio: 0.5 (95% CI 0.4–0.8)	Age, sex, educational level, disease duration, and disease activity

## References

[1] Chorus A.M., Miedema H.S., Wevers C.W., van der Linden S. (2001). Work factors and behavioural coping in relation to withdrawal from the labour force in patients with rheumatoid arthritis. Ann. Rheum. Dis..

[2] Holma I.A., Holma K.M., Melartin T.K., Rytsala H.J., Isometsa E.T. (2012). A 5-year prospective study of predictors for disability pension among patients with major depressive disorder. Acta Psychiatr. Scand..

[3] Burmeister E.A., Kalisch B.J., Xie B., Doumit M.A.A., Lee E., Ferraresion A., Terzioglu F., Bragadottir H. (2019). Determinants of nurse absenteeism and intent to leave: An international study. J. Nurs. Manag..

[4] Thern E., Falkstedt D., Almroth M., Kjellberg K., Landberg J., Bodin T., Melin B., Hemmingsson T. (2022). Educational qualification differences and early labor market exit among men: The contribution of labor market marginalization measured across the working life. BMC Public Health.

[5] Liu J., Mao Y. (2020). Continuing medical education and work commitment among rural healthcare workers: A cross-sectional study in 11 western provinces in China. BMJ Open.

[6] Moloney W., Boxall P., Parsons M., Cheung G. (2018). Factors predicting Registered Nurses’ intentions to leave their organization and profession: A job demands-resources framework. J. Adv. Nurs..

[7] Turner S., Ross M.K., Ibbetson R.J. (2012). The impact of General Dental Council registration and continuing professional development on UK dental care professionals: (1) dental nurses. Br. Dent. J..

[8] Knardahl S., Johannessen H.A., Sterud T., Härmä M., Rugulies R., Seitsamo J., Borg V. (2017). The contribution from psychological, social, and organizational work factors to risk of disability retirement: A systematic review with meta-analyses. BMC Public Health.

[9] Allen L.M., Palermo C., Armstrong E., Hay M. (2019). Categorising the broad impacts of continuing professional development: A scoping review. Med. Educ..

[10] Beckman D., Wardian J., Sauerwein T.J., True M.W. (2019). Evaluation of an interprofessional continuing professional development course on comprehensive diabetes care: A mixed-methods approach. J. Eval. Clin. Pract..

[11] Smith J., Kean S., Vauhkonen A., Elonen I., Silva S.C., Pajari J., Cassar M., Martin-Delgado L., Zrubcova D., Salminen L. (2023). An integrative review of the continuing professional development needs for nurse educators. Nurse Educ. Today.

[12] Robinson S., Tingle A. (2003). Continuing education opportunities for recently qualified mental health diplomates. J. Psychiatr. Ment. Health Nurs..

[13] Phillips J.L., Heneka N., Bhattarai P., Fraser C., Shaw T. (2019). Effectiveness of the spaced education pedagogy for clinicians’ continuing professional development: A systematic review. Med. Educ..

[14] Haywood H., Pain H., Ryan S., Adams J. (2013). The continuing professional development for nurses and allied health professionals working within musculoskeletal services: A national UK survey. Musculoskelet. Care.

[15] Katona C., Bíró É., Vincze S., Kósa K. (2022). On-the-job vocational training of nonprofessional ethnic health workers of a primary health care team improves their sense of coherence. Hum. Resour. Health.

[16] Chenoweth L., Merlyn T., Jeon Y.H., Tait F., Duffield C. (2014). Attracting and retaining qualified nurses in aged and dementia care: Outcomes from an Australian study. J. Nurs. Manag..

[17] Cross W., Wyman P.A. (2006). Training and motivational factors as predictors of job satisfaction and anticipated job retention among implementers of a school-based prevention program. J. Prim. Prev..

[18] Leppel K., Brucker E., Cochran J. (2012). The importance of job training to job satisfaction of older workers. J. Aging Soc. Policy..

[19] Robertson E.M., Higgins L., Rozmus C., Robinson J.P. (1999). Association between continuing education and job satisfaction of nurses employed in long-term care facilities. J. Contin. Educ. Nurs..

[20] Moher D., Liberati A., Tetzlaff J., Altman D.G., Group P. (2009). Preferred reporting items for systematic reviews and meta-analyses: The PRISMA statement. Ann. Intern. Med..

[21] Armijo-Olivo S., Stiles C.R., Hagen N.A., Biondo P.D., Cummings G.G. (2012). Assessment of study quality for systematic reviews: A comparison of the Cochrane Collaboration Risk of Bias Tool and the Effective Public Health Practice Project Quality Assessment Tool: Methodological research. J. Eval. Clin. Pract..

[22] Gallego G., Dew A., Lincoln M., Bundy A., Chedid R.J., Bulkeley K., Brentnall J., Veitch C. (2015). Should I stay or should I go? Exploring the job preferences of allied health professionals working with people with disability in rural Australia. Hum. Resour. Health.

[23] Garrett T. (2008). Pharmacy workforce recruitment and retention: An Australian Area Health Service perspective. J. Pharm. Pract. Res..

[24] Gan Y., Jiang H., Li L., Yang Y., Wang C., Liu J., Yang T., Zheng Y., Zhu Y., Sampson O. (2020). A national survey of turnover intention among general practitioners in China. Int. J. Health Plann. Manag..

[25] Wang X., Qin H., Zhu Y., Wang Z., Ye B., Zhu X., Liang Y. (2022). Association of off-the-job training with work performance and work-family conflict among physicians: A cross-sectional study in China. BMJ Open.

[26] Lau T., Kumar S., Robinson E. (2004). New Zealand’s psychiatrist workforce: Profile, recruitment and retention. Aust. N. Z. J. Psychiatry.

[27] Chang S., Morahan P.S., Magrane D., Helitzer D., Lee H.Y., Newbill S., Peng H.L., Guindani M., Cardinali G. (2016). Retaining Faculty in Academic Medicine: The Impact of Career Development Programs for Women. J. Womens Health.

[28] Ries A., Wingard D., Gamst A., Larsen C., Farrell E., Reznik V. (2012). Measuring faculty retention and success in academic medicine. Acad. Med..

[29] Nowrouzi B., Rukholm E., Lariviere M., Carter L., Koren I., Mian O., Giddens E. (2016). An examination of retention factors among registered nurses in Northeastern Ontario, Canada: Nurses intent to stay in their current position. Work.

[30] Holm A., Høgelund J., Gørtz M., Rasmussen K.S., Houlberg H.S. (2017). Employment effects of active labor market programs for sick-listed workers. J. Health Econ..

[31] Kols A., Kibwana S., Molla Y., Ayalew F., Teshome M., van Roosmalen J., Stekelenburg J. (2018). Factors Predicting Ethiopian Anesthetists’ Intention to Leave Their Job. World J. Surg..

[32] Flinkman M., Laine M., Leino-Kilpi H., Hasselhorn H.M., Salantera S. (2008). Explaining young registered Finnish nurses’ intention to leave the profession: A questionnaire survey. Int. J. Nurs. Stud..

[33] Agyapong V.I., Osei A., Farren C.K., McAuliffe E. (2015). Factors influencing the career choice and retention of community mental health workers in Ghana. Hum. Resour. Health.

[34] Miura H., Tano R., Oshima K., Usui Y. (2021). Analysis of Factors Related to Working Status of Dental Hygienists in Japan. Int. J. Environ. Res. Public Health.

[35] Tomietto M., Rappagliosi C.M., Sartori R., Battistelli A. (2015). Newcomer nurses’ organisational socialisation and turnover intention during the first 2 years of employment. J. Nurs. Manag..

[36] Malik O.F., Abbas Q., Kiyani T.M., Malik K., Waheed A. (2011). Perceived investment in employee development and turnover intention: A social exchange perspective. Afr. J. Bus. Manag..

[37] Yu M., Kang K.J. (2016). Factors Affecting Turnover Intention for New Graduate Nurses in Three Transition Periods for Job and Work Environment Satisfaction. J. Contin. Educ. Nurs..

[38] Fochsen G., Sjogren K., Josephson M., Lagerstrom M. (2005). Factors contributing to the decision to leave nursing care: A study among Swedish nursing personnel. J. Nurs. Manag..

[39] Du P., Huang I., Huang Y., Chang C. (2019). Dual normative commitments mediating the relationship between perceived investment in employees’ development and intention to leave among the healthcare workforce in underserviced areas of Taiwan. Rural Remote Health.

[40] Lee C.H., Bruvold N.T. (2003). Creating value for employees: Investment in employee development. Int. J. Hum. Resour. Manag..

[41] Estryn-Behar M., van der Heijden B.I., Fry C., Hasselhorn H.M. (2010). Longitudinal analysis of personal and work-related factors associated with turnover among nurses. Nurs. Res..

[42] Marinucci F., Majigo M., Wattleworth M., Paterniti A.D., Hossain M.B., Redfield R. (2013). Factors affecting job satisfaction and retention of medical laboratory professionals in seven countries of Sub-Saharan Africa. Hum. Resour. Health.

[43] Markowski M., Cleaver K., Weldon S.M. (2020). An integrative review of the factors influencing older nurses’ timing of retirement. J. Adv. Nurs..

[44] Andrews J., Manthorpe J., Watson R. (2005). Employment transitions for older nurses: A qualitative study. J. Adv. Nurs..

[45] Ahmad K.Z., Abu Bakar R. (2003). The association between training and organizational commitment among white-collar workers in Malaysia. Int. J. Train. Dev..

[46] Rimsza M.E., Ruch-Ross H., Simon H.K., Pendergass T.W., Mulvey H.J. (2017). Factors Influencing Pediatrician Retirement: A Survey of American Academy of Pediatrics Chapter Members. J. Pediatr..

[47] Ifeoma E.A., Rebecca E.F., Ezekiel O.O., Mary-Jane O., Uyime E.C., Obiageri E.N., Tola A., James D. (2015). A cross-sectional study of the knowledge and attitude of medical laboratory personnel regarding continuing professional development. Niger. Med. J..

[48] Alameddine M., Baumann A., Laporte A., Deber R. (2012). A narrative review on the effect of economic downturns on the nursing labour market: Implications for policy and planning. Hum. Resour. Health.

[49] Smith T., Fisher K., Keane S., Lincoln M. (2011). Comparison of the results of two rural allied health workforce surveys in the Hunter New England region of New South Wales: 2005 versus 2008. Aust. J. Rural Health.

[50] Decius J., Knappstein M., Klug K. (2023). Which way of learning benefits your career? The role of different forms of work-related learning for different types of perceived employability. Eur. J. Work. Organ. Psychol..

