# Supplementary material for: The Role of Continuing Professional Training or Development in Maintaining Current Employment: A Systematic Review
Source: Healthcare (Basel). 2023 Nov 3;11(21):2900. doi: 10.3390/healthcare11212900 (PMC10647344; doi:10.3390/healthcare11212900)
Supplement: Supplementary file 1 [file healthcare-11-02900-s001.zip › healthcare-2602781-supplementary.pdf]

Supplemental Tables

Supplemental Table S1. Adapted Effective Public Health Practice Project quality assessment.

Type of domain	Criteria definition	Classification (potential for bias)
Selection bias	Sampling method of the study population, representativeness (response rate, difference between responders and non-responders, investigation, and control of variables in case of difference between responders and non-responders	**Weak:** Target population defined as representative of the general population or subgroup of the general population (specific age group, women, men, specific geographic area, and specific occupational group) and response rate is above 80%.<br>**Moderate:** Target population defined as somewhat representative of the general population, a restricted subgroup of the general population, response rate 60%-79%.<br>**Strong:** Target population defined as "self-referred"/ volunteers, response rate less than 60%.
Performance bias	Valid and reliable assessment of exposure<br>Assessors blinded for outcome status	**Weak:** Register data or detail information on duration, type, and content of training.<br>**Moderate:** A dichotomized (yes/no) self-reported question was used.
Detection bias	Clear definition of outcome<br>Standard method for outcome assessment<br>Assessor of outcome blinded to exposure status	**Weak:** The outcome assessment was based on register data.<br>**Moderate:** The outcome assessment was based on self-reported measures.
Confounding	Matching<br>Stratification<br>Statistical analysis	**Weak:** Considered confounders and controlled for 80%-100% of confounders.<br>**Moderate:** Considered confounders and controlled for 60%-79%.<br>**Strong:** Considered confounders and controlled for less than 60%.
Attrition bias	Withdrawal and drop- out rates<br>Size of missing data	**Weak:** Follow up participation rate of more than 80%, or missing data of less than 20%.<br>**Moderate:** Follow up participation rate of 60%-79%, or missing data of 20%-40%.<br>**Strong:** Follow up participation rate of less than 60%, or missing data of more than 40%.

Supplemental Table S2. Risk of bias assessment

Study (authors, year of publication and country)	Selection	Performance	Detection	Attrition	Confounding
**Cohort studies**					
Holm et al. 2017, Denmark [1]	Low	Moderate	Low	Low	Low
Chang et al. 2016, USA [2]	Low	Low	Low	Low	Moderate
Ries et al. 2012, USA [3]	Moderate	Low	Low	Low	Moderate
Robinson and Tingle 2003, UK [4]	Low	Moderate	Moderate	Moderate	High
**Cross-sectional studies**					
Wang et al. 2022, China [5]	High	Low	Moderate	Low	Low
Miura et al. 2021, Japan [6]	High	Moderate	Moderate	Low	High
Gan et al. 2020, China [7]	Low	Moderate	Moderate	Low	Low
Liu & Mao 2020, China [8]	Low	Moderate	Moderate	Low	Low
Du et al. 2019, Taiwan [9]	Low	Moderate	Moderate	Low	Moderate
Kols et al. 2018, Ethiopia [10]	Moderate	Moderate	Moderate	Low	Moderate
Moloney et al. 2018, New Zealand [11]	High	Moderate	Moderate	Low	Moderate
Nowrouzi et al. 2016, Canada [12]	High	Moderate	Moderate	Low	Low
Yu and Kang 2016, South Korea [13]	Moderate	Moderate	Moderate	Low	Low
Agyapong et al. 2015, Ghana [14]	Moderate	Moderate	Moderate	Low	High
Gallego et al. 2015, Australia [15]	High	Moderate	Moderate	Low	Moderate
Tomietto et al. 2015, Italy [16]	Moderate	Moderate	Moderate	Low	Moderate
Chenoweth et al. 2013, Australia [17]	High	Moderate	Moderate	Low	High
Marinucci et al. 2013, 7 Sub-Saharan Africa countries [18]	High	Moderate	Moderate	Low	High
Turner et al. 2012, UK [19]	High	Moderate	Moderate	Low	Low
Malik et al. 2011, Pakistan [20]	Moderate	Moderate	Moderate	Low	Moderate
Estryn-Behar et al. 2010 [21]	High	Moderate	Moderate	High	High
Flinkman et al. 2008 [22]	Moderate	Moderate	Moderate	Low	High
Garrett et al. 2008, Australia [23]	High	Moderate	Moderate	Low	High
Fochsen et al. 2005, Sweden [24]	Moderate	Moderate	Moderate	Low	Moderate
Lau et al. 2004, New Zealand [25]	Moderate	Moderate	Moderate	Low	High
Lee and Bruvold 2003, Singapore and USA [26]	Moderate (Low for sample from Singapore and high for sample from USA)	Moderate	Moderate	Low	Moderate
Chorus et al. 2001, the Netherlands [27]	Moderate	Moderate	Moderate	Low	Moderate

References

1. Holm, A.; Høgelund, J.; Gørtz, M.; Rasmussen, K.S.; Houlberg, H.S. Employment effects of active labor market programs for sick-listed workers. *J. Health Econ.* **2017**, *52*, 33–44. https://doi.org/10.1016/j.jhealeco.2017.01.006.
2. Chang, S.; Morahan, P.S.; Magrane, D.; Helitzer, D.; Lee, H.Y.; Newbill, S.; Peng, H.L.; Guindani, M.; Cardinali, G. Retaining Faculty in Academic Medicine: The Impact of Career Development Programs for Women. *J. Womens Health* **2016**, *25*, 687–696. https://doi.org/10.1089/jwh.2015.5608.
3. Ries, A.; Wingard, D.; Gamst, A.; Larsen, C.; Farrell, E.; Reznik, V. Measuring faculty retention and success in academic medicine. *Acad. Med.* **2012**, *87*, 1046–1051. https://doi.org/10.1097/ACM.0b013e31825d0d31.
4. Robinson, S.; Tingle, A. Continuing education opportunities for recently qualified mental health diplomates. *J. Psychiatr. Ment. Health Nurs.* **2003**, *10*, 659–668. https://doi.org/10.1046/j.1365-2850.2003.00648.x.
5. Wang, X.; Qin, H.; Zhu, Y.; Wang, Z.; Ye, B.; Zhu, X.; Liang, Y. Association of off-the-job training with work performance and work-family conflict among physicians: A cross-sectional study in China. *BMJ Open* **2022**, *12*, e053280. https://doi.org/10.1136/bmjopen-2021-053280.
6. Miura, H.; Tano, R.; Oshima, K.; Usui, Y. Analysis of Factors Related to Working Status of Dental Hygienists in Japan. *Int. J. Environ. Res. Public Health* **2021**, *18*, 1025. https://doi.org/10.3390/ijerph18031025.
7. Gan, Y.; Jiang, H.; Li, L.; Yang, Y.; Wang, C.; Liu, J.; Yang, T.; Zheng, Y.; Zhu, Y.; Sampson, O.; et al. A national survey of turnover intention among general practitioners in China. *Int. J. Health Plann. Manag.* **2020**, *35*, 482–493. https://doi.org/10.1002/hpm.2921.

8. Liu, J.; Mao, Y. Continuing medical education and work commitment among rural healthcare workers: A cross-sectional study in 11 western provinces in China. *BMJ Open* **2020**, *10*, e037985. https://doi.org/10.1136/bmjopen-2020-037985.
9. Du, P.; Huang, I.; Huang, Y.; Chang, C. Dual normative commitments mediating the relationship between perceived investment in employees' development and intention to leave among the healthcare workforce in underserviced areas of Taiwan. *Rural Remote Health* **2019**, *19*, 4837. https://doi.org/10.22605/RRH4837.
10. Kols, A.; Kibwana, S.; Molla, Y.; Ayalew, F.; Teshome, M.; van Roosmalen, J.; Stekelenburg, J. Factors Predicting Ethiopian Anesthetists' Intention to Leave Their Job. *World J. Surg.* **2018**, *42*, 1262–1269. https://doi.org/10.1007/s00268-017-4318-7.
11. Moloney, W.; Boxall, P.; Parsons, M.; Cheung, G. Factors predicting Registered Nurses' intentions to leave their organization and profession: A job demands-resources framework. *J. Adv. Nurs.* **2018**, *74*, 864–875. https://doi.org/10.1111/jan.13497.
12. Nowrouzi, B.; Rukholm, E.; Lariviere, M.; Carter, L.; Koren, I.; Mian, O.; Giddens, E. An examination of retention factors among registered nurses in Northeastern Ontario, Canada: Nurses intent to stay in their current position. *Work* **2016**, *54*, 51–58. https://doi.org/10.3233/WOR-162267.
13. Yu, M.; Kang, K.J. Factors Affecting Turnover Intention for New Graduate Nurses in Three Transition Periods for Job and Work Environment Satisfaction. *J. Contin. Educ. Nurs.* **2016**, *47*, 120–131. https://doi.org/10.3928/00220124-20160218-08.
14. Agyapong, V.I.; Osei, A.; Farren, C.K.; McAuliffe, E. Factors influencing the career choice and retention of community mental health workers in Ghana. *Hum. Resour. Health* **2015**, *13*, 56. https://doi.org/10.1186/s12960-015-0050-2.
15. Gallego, G.; Dew, A.; Lincoln, M.; Bundy, A.; Chedid, R.J.; Bulkeley, K.; Brentnall, J.; Veitch, C. Should I stay or should I go? Exploring the job preferences of allied health professionals working with people with disability in rural Australia. *Hum. Resour. Health* **2015**, *13*, 53. https://doi.org/10.1186/s12960-015-0047-x.
16. Tomietto, M.; Rappagliosi, C.M.; Sartori, R.; Battistelli, A. Newcomer nurses' organisational socialisation and turnover intention during the first 2 years of employment. *J. Nurs. Manag.* **2015**, *23*, 851–858. https://doi.org/10.1111/jonm.12224.
17. Chenoweth, L.; Merlyn, T.; Jeon, Y.H.; Tait, F.; Duffield, C. Attracting and retaining qualified nurses in aged and dementia care: Outcomes from an Australian study. *J. Nurs. Manag.* **2014**, *22*, 234–247. https://doi.org/10.1111/jonm.12040.
18. Marinucci, F.; Majigo, M.; Wattleworth, M.; Paterniti, A.D.; Hossain, M.B.; Redfield, R. Factors affecting job satisfaction and retention of medical laboratory professionals in seven countries of Sub-Saharan Africa. *Hum. Resour. Health* **2013**, *11*, 38. https://doi.org/10.1186/1478-4491-11-38.
19. Turner, S.; Ross, M.K.; Ibbetson, R.J. The impact of General Dental Council registration and continuing professional development on UK dental care professionals: (1) dental nurses. *Br. Dent. J.* **2012**, *213*, E2. https://doi.org/10.1038/sj.bdj.2012.664.
20. Malik, O.F.; Abbas, Q.; Kiyani, T.M.; Malik, K.; Waheed, A. Perceived investment in employee development and turnover intention: A social exchange perspective. *Afr. J. Bus. Manag.* **2011**, *5*, 1904–1914. https://doi.org/10.5897/AJBM10.1024.
21. Estryn-Behar, M.; van der Heijden, B.I.; Fry, C.; Hasselhorn, H.M. Longitudinal analysis of personal and work-related factors associated with turnover among nurses. *Nurs. Res.* **2010**, *59*, 166–177. https://doi.org/10.1097/NNR.0b013e3181dbb29f.
22. Flinkman, M.; Laine, M.; Leino-Kilpi, H.; Hasselhorn, H.M.; Salantera, S. Explaining young registered Finnish nurses' intention to leave the profession: A questionnaire survey. *Int. J. Nurs. Stud.* **2008**, *45*, 727–739. https://doi.org/10.1016/j.ijnurstu.2006.12.006.
23. Garrett, T. Pharmacy workforce recruitment and retention: An Australian Area Health Service perspective. *J. Pharm. Pract. Res.* **2008**, *38*, 183–187.
24. Fochsen, G.; Sjogren, K.; Josephson, M.; Lagerstrom, M. Factors contributing to the decision to leave nursing care: A study among Swedish nursing personnel. *J. Nurs. Manag.* **2005**, *13*, 338–344. https://doi.org/10.1111/j.1365-2934.2005.00546.x.
25. Lau, T.; Kumar, S.; Robinson, E. New Zealand's psychiatrist workforce: Profile, recruitment and retention. *Aust. N. Z. J. Psychiatry* **2004**, *38*, 547–553. https://doi.org/10.1080/j.1440-1614.2004.01408.x.
26. Lee, C.H.; Bruvold, N.T. Creating value for employees: Investment in employee development. *Int. J. Hum. Resour. Manag.* **2003**, *14*, 981–1000.
27. Chorus, A.M.; Miedema, H.S.; Wevers, C.W.; van der Linden, S. Work factors and behavioural coping in relation to withdrawal from the labour force in patients with rheumatoid arthritis. *Ann. Rheum. Dis.* **2001**, *60*, 1025–1032. https://doi.org/10.1136/ard.60.11.1025.
